# Foxp3 and IL-10 Expression Correlates with Parasite Burden in Lesional Tissues of Post Kala Azar Dermal Leishmaniasis (PKDL) Patients

**DOI:** 10.1371/journal.pntd.0001171

**Published:** 2011-05-31

**Authors:** Gajendra Kumar Katara, Nasim Akhtar Ansari, Sandeep Verma, V. Ramesh, Poonam Salotra

**Affiliations:** 1 Institute of Pathology (ICMR), Safdarjung Hospital Campus, New Delhi, India; 2 Department of Dermatology, Safdarjung Hospital, New Delhi, India; Queensland Institute of Medical Research, Australia

## Abstract

**Background:**

Post kala-azar dermal leishmaniasis (PKDL), a sequel to visceral leishamaniasis (VL) in 5–15% cases, constitutes a parasite reservoir important in disease transmission. The precise immunological cause of PKDL outcome remains obscure. However, overlapping counter regulatory responses with elevated IFN-γ and IL-10 are reported.

**Methodology/Principal Findings:**

Present study deals with ex-vivo mRNA and protein analysis of natural regulatory T cells (nTreg) markers (Foxp3, CD25 and CTLA-4) and IL-10 levels in lesion tissues of PKDL patients at pre and post treatment stages. In addition, correlation of nTreg markers and IL-10 with parasite load in tissue lesions was investigated. mRNA levels of nTreg markers and IL-10 were found significantly elevated in pre-treatment PKDL cases compared to controls (Foxp3, P = 0.0009; CD25 & CTLA-4, P<0.0001; IL-10, P<0.0001), and were restored after treatment. Analysis of nTreg cell markers and IL-10 in different clinical manifestations of disease revealed elevated levels in nodular lesions compared to macules/papules. Further, Foxp3, CD25 and IL-10 mRNA levels directly correlated with parasite load in lesions tissues.

**Conclusion/Significance:**

Data demonstrated accumulation of nTreg cells in infected tissue and a correlation of both IL-10 and nTreg levels with parasite burden suggesting their role in disease severity in PKDL.

## Author Summary

Post kala azar dermal leishamniasis (PKDL), an unusual dermatosis develops in 5–15% of apparently cured visceral leishmaniasis cases in India and in about 60% of cases in Sudan. PKDL cases assume importance since they constitute a major human reservoir for the parasite. Inadequate treatment of VL, genetics, nutrition and immunological mechanisms that allow renewed multiplication of latent parasites or reinfection predispose to PKDL. Immunopathogenesis of PKDL is poorly understood. IL-10 is widely accepted as an immuno-suppressive cytokine and produced by diverse cell populations including, B cells, macrophages and CD4+ T cells. Natural T regulatory (nTreg) cells are subpopulation of CD4+ T cells that inhibit the response of other T cells. In this study we reported the accumulation of nTreg cells in lesion tissues of PKDL patients. Further correlation of Treg markers and IL-10 with parasite load in lesion tissues suggested a role of IL-10 and Treg in parasite establishment or persistence. Further studies are warranted to explore antigen specific IL-10 source in lesion tissues and unravel the concerted induction or accumulation of Treg in PKDL.

## Introduction

Leishmaniasis constitutes various forms of globally widespread group of neglected diseases caused by an obligatory intracellular protozoan parasite of genus *Leishmania*. It is currently endemic in 88 countries and overall prevalence is estimated as 12 million with 350 million at risk. Visceral leishmaniasis (VL) is the most severe form, fatal if not treated. 90% of all VL cases world wide occur in India, Bangladesh, Nepal, Brazil and Sudan [1]. In India, *Leishmania donovani* causes VL or Kala azar (KA) and Post kala azar dermal leishmaniasis (PKDL) while *L. tropica* is responsible for cutaneous leishmaniasis (CL) in humans.

PKDL is an unusual dermatosis that develops in 5–15% of apparently cured VL cases in India and in about 60% of cases in Sudan [2]. This chronic skin condition produces gross cutaneous lesions in the form of hypopigmented macules, erythema and nodular stages. So far, little is known about the parasite/host factors that drive the parasite to shift from site of initial infection viscera (spleen or bone marrow) to the dermis or about the clinical manifestation of the disease. Inadequate treatment is considered to be a factor in PKDL development; however, the disease may develop even after complete treatment. Factors such as genetics and nutrition may be important [2] and remain to be explored in Indian PKDL.

The precise immunological cause remains obscure. In Sudanese PKDL, immune suppression, reinfection or reactivation is considered to be the major underlying cause of PKDL development [2]. Reactivation of disease in the form of PKDL is suggested, on account of retention and maintenance of residual IL-10 and TGF-β levels in sodium antimony gluconate (SAG) treated KA individuals [3]. However, current reports suggest that PKDL may develop even after treatment with anti-leishmanial drugs such as Amphotericin B or Miltefosine. Thus, other mechanisms may be responsible for disease development.

Like human VL, elevated levels of IFN-γ and TNF-α are reported systemically or in lesion tissues of PKDL with simultaneous presence of immunosuppressive cytokine, IL-10, suggesting that there is no defect in mounting antigen specific responses [4]. Direct correlation between circulating IL-10 levels with parasite load suggests role of IL-10 in compromising the effector T cell function in human VL [5], [6]. In, addition IL-10 knockout mice are highly resistant to *L. donovani* infection and treatment with anti-IL-10 receptor antibody promotes clinical cure [7].

Several IL-10–producing CD4^+^ T cell subpopulations have been described, among them naturally occurring CD4^+^CD25^+^Foxp3^+^ regulatory T (nTreg) cells are one such sub-population with unique ability to inhibit the response of other T cells. It is characterized by the constitutive expression of IL-2R-α chain (CD25) and by expression of the transcriptional factor, Foxp3. Evidence from experimental murine models of *L. major* infection suggests that nTreg cells promote survival of *Leishmania* parasites and reactivation of disease [8]. In addition, in human CL intralesional nTreg have been associated with SAG unresponsiveness and disease pathology [9], [10].

In our previous report we proposed the role of nTreg in PKDL in the context of overlapping counter-regulating cytokines and chronic persistent infection [4]. The present study was carried out to evaluate if nTreg and IL-10 have an influence on disease persistence in human. The study supports the role of IL-10 and nTreg in PKDL pathology, as evident from a direct correlation of IL-10 and Foxp3 mRNA levels with parasite load within lesion tissues.

## Materials and Methods

### Study subject and Tissue samples

Lesional skin tissues were collected from PKDL patients (n = 25) originating from Bihar and reporting to the Department of Dermatology, Safdarjung Hospital, New Delhi (Table 1). Biopsies were collected from face (n = 19) or shoulder region (n = 6). All patients were HIV seronegative and diagnosis was confirmed by microscopy and polymerase chain reaction, described previously [4]. Furthermore, based on lesion types, PKDL patients were categorized in 3 groups, nodular (N) (n = 12), macular or papular (M/P) (n = 10) and polymorphic (n = 3). Patients were treated with oral Miltefosine (150 mg/day) for 2 months. Follow-up samples were collected from the same site as at pre treatment stage one month after completion of treatment in 8 cases, all of which showed apparent clinical cure. Further no parasites were detectable by real time PCR in any of these cases at this stage. 5 normal skin tissues were collected from the shoulder region of healthy individuals.

**Table 1:** Major characteristics of the study population.

Patients Characteristics	PKDL(n = 25)
**Age (years) range, (mean±SD)**	19–52, (27.72±8.48)
**Sex (M/F)**	22/3
**Cases reporting history of KA**	19
**History of KA, range in years, (mean±SD)**	2–34, (10.57±8.89)
**Duration of PKDL, range in years, (mean±SD)**	0.5–31, (5.2±6.85)
**Type of lesions**	
Nodular	12
Macular/Papular	10
Polymorphic	3

Abbreviations: M = Male, F = Female, PKDL = Post kala azar dermal leishmaniasis, KA = Kala azar.

### Ethics Statement

The study was approved by and carried out under the guidelines of the Ethical Committee of the Safdarjung Hospital, India. All patients provided written informed consent for the collection of samples and subsequent analysis.

### Quantitative real times reverse transcription PCR

Punch biopsy (4–6 mm) samples were collected from PKDL patients and healthy individuals in RNA*later* (Ambion, Austin, TX, USA) and stored in liquid nitrogen until use. Total RNA was isolated using Trizol reagent, in accordance with the manufacturer's instructions, and quality of RNA was assessed using Bioanalyzer (Agilent, Foster City, CA, USA). Quantity of RNA was determined by Nanodrop spectrophotometer (Thermo Scientific, Wilmington, DE, USA). cDNA was synthesized from 2 µg of total RNA using High capacity cDNA synthesis kit (Applied Biosystems, Foster City, CA, USA). Incubation conditions for reverse transcription were 10 min at 25°C, followed by 2 hours at 37°C and were performed on a MasterCycler Gradient (Eppendorf, Hamburg Germany).

Real-time polymerase chain reaction was performed on an ABI Prism 7000 sequence detection system (Applied Biosystems) using cDNA specific FAM-MGB–labeled Taqman primer/probe sets (Applied Biosystems) for IL-10 (Hs00174086_m1), CD25 (Hs00166229_m1), CTLA-4 (Hs00175480_m1), Foxp3 (Hs00203958_m1). VIC-MGB labeled 18S rRNA (4319413E) was used as endogenous control for relative amount of mRNA in each sample. The relative quantification of products was determined by the number of cycles over endogenous control required to detect the gene expression of interest.

### Quantification of parasite load

PKDL skin lesion tissue (n = 12) was collected in NET buffer [150 mM NaCl, 15 mM Tris-HCl (pH 8.3) and 1 mM EDTA]. Tissue was homogenized in liquid nitrogen and DNA was extracted using QIAamp DNA Tissue kit (QIAGEN) according to manufacturer's instructions. All samples were processed on the same day and isolated DNA was stored at −80°C until use. DNA samples from 3 patients who were part of our previous study [5] were also included. SYBR Green I based Real-time PCR was used for accurate quantification of parasite load as described previously [5]. Briefly, PCR reaction was performed in an ABI Prism 7000 sequence detection system (Applied Biosystems) using forward primer (5′-CTTTTCTGGTCCTCCGGGTAGG-3′), reverse primer, (5′-CCACCCGGCCCTATTTTACACCAA-3′). A standard curve was constructed using 10-fold serially diluted *L. donovani* parasite DNA corresponding to 10^4^ to 0.1 parasite per reaction.

### Immunohistochemistry (IHC)

Punch biopsy skin tissue was collected in neutralized formaline. The tissue was paraffin embedded and 5 µm sections were prepared on polylysine coated glass slides from all skin specimens. Immunohistochemical staining was performed using a standard polymer-peroxidase technique (Dako, Carpinteria, CA, USA). After deparaffination, rehydration and blockade of endogenous peroxidase activity, heat-induced antigen retrieval was performed in Tris-EDTA buffer (pH 9.0). After antigen retrieval sections were covered with serum-free protein block (Dako) for 1 hr, followed by incubation with anti-human Foxp3 (e-biosciences, San Diego, USA) for 1 hr and EnVision1 anti-mouse/horseradish peroxidase (HRP) polymer (Dako) for 30 min at room temperature. Color was developed using diaminobenzidine (DAB1) chromogen system.

### Statistical analysis

Statistical analysis was performed using Graph Pad Prism 5 (GraphPad Software, Inc., San Diego, CA, USA). Correlation was evaluated using Spearman/Pearson correlation test. *P* values of less than 0.05 were considered significant.

## Results

### Localized levels of Treg cell markers and IL-10 increased in PKDL

**Figure 1:**
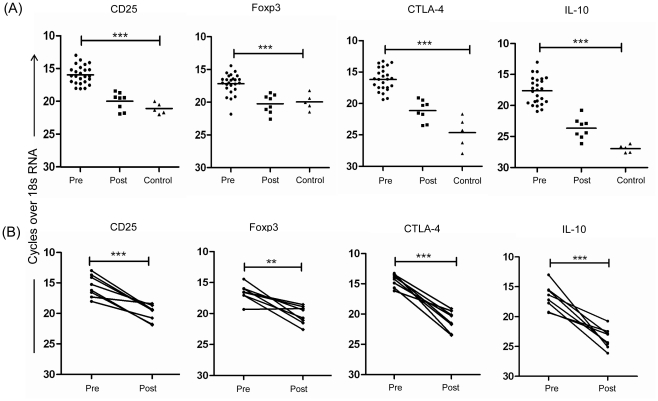
*Ex vivo* analysis of mRNA expression of Treg markers and IL-10 in PKDL. Relative mRNA levels of CD25, Foxp3, CTLA-4 and IL-10 in lesion tissues of PKDL patients determined by real time polymerase chain reaction at pretreatment (n = 25) or post treatment (n = 8), or control tissues (n = 5) (A) and in paired samples (n = 8) shown separately (B). **P<0.01, and ***P<0.001.

Natural Treg markers and IL-10 mRNA level were evaluated in skin lesion tissues of PKDL patients and compared with healthy controls tissues. mRNA analysis showed significantly elevated levels of nTreg markers in pre-treatment cases compared to control (Foxp3, *P* = 0.0009; CD25 & CTLA-4, *P*<0.0001) (Figure 1A). After treatment, a significant decrement in mRNA levels (Foxp3, *P* = 0.0025; CD25, *P* = 0.0002 & CTLA-4, *P*<0.0001) was noticed in paired samples (Figure 1B). In addition, we evaluated mRNA levels of IL-10, a molecule produced by adaptive Treg or Tr1 cells, and frequently associated with experimental or human VL pathology [5]–[7]. IL-10 mRNA level was significantly elevated in PKDL compared to control (*P*<0.0001). A significant drop in IL-10 mRNA levels was noticed in paired samples (*P* = 0.0004) (Figure 1).

### Intralesional expression of CD25, Foxp3 and IL-10 correlated with parasite load

**Figure 2:**
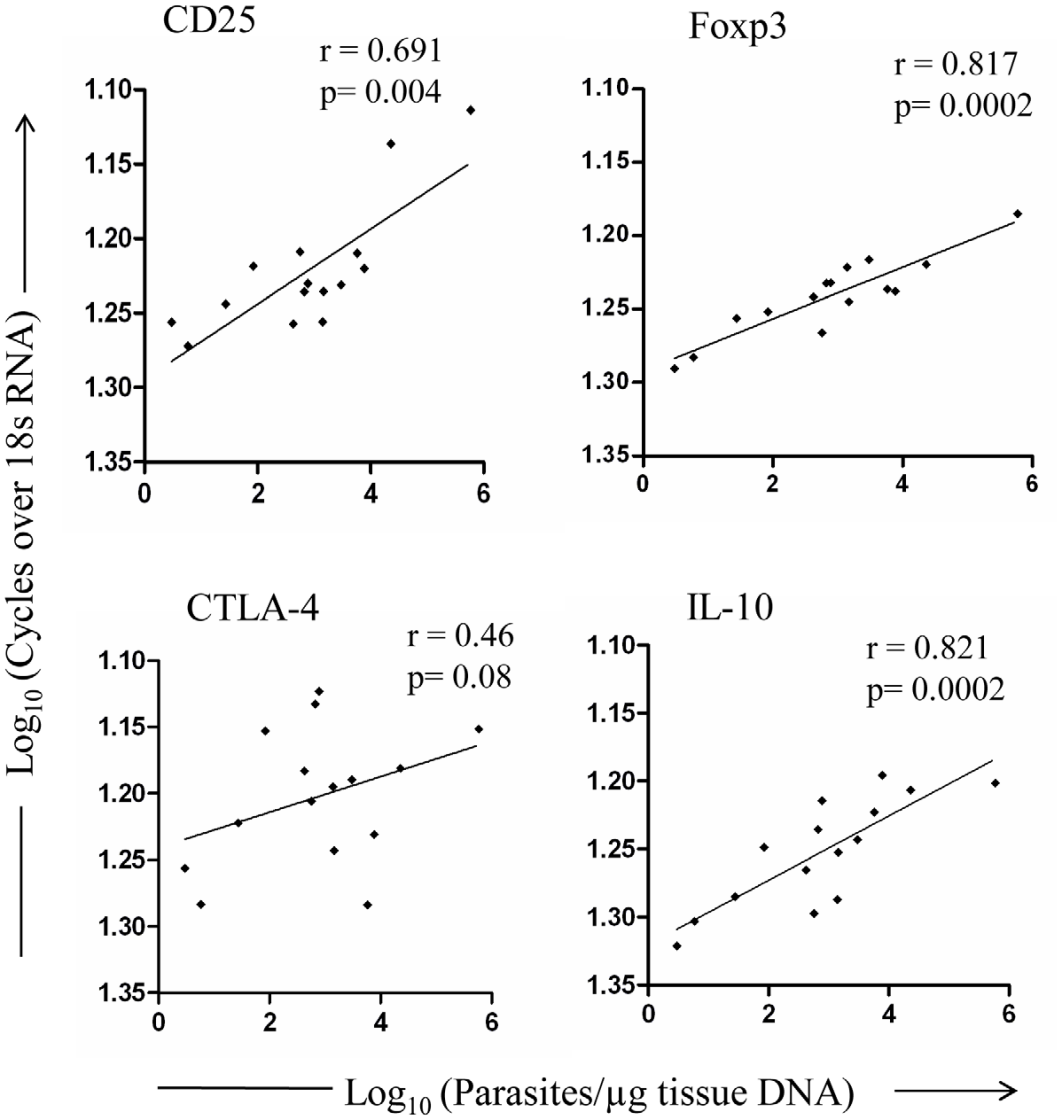
Correlation of mRNA expression of Treg markers and IL-10 with parasite load in PKDL. Relative mRNA levels of Treg markers and IL-10 with respect to parasite load within lesion tissues of PKDL patients (n = 15). Plot was created using logarithmic values for cycles over 18sRNA and parasite load. Correlation was calculated using Spearman/Pearson correlation test. Diagonal line represents linear regression.

To further evaluate whether there is any direct association between expression of these molecules and parasite burden, we evaluated the parasite load in PKDL lesion tissues (n = 15). The median parasite load was 776 parasites/µg tissue DNA (range = 3 to 590,000 parasites/µg tissue DNA), with a higher parasite load in nodular lesions (median 2,244 parasites/µg tissue DNA, n = 10) as compared to that in papular/macular lesions (median 28 parasites/µg tissue DNA, n = 5). *Ex vivo* analysis showed a direct correlation between parasite load and mRNA levels in lesion tissues (CD25, r = 0.691; Foxp3, r = 0.817; IL-10, r = 0.821), such correlation was not noticed for CTLA-4 (Figure 2). No parasite was detected in any of the post treatment sample.

### Aggregation of Foxp3+ cells in PKDL decreased with treatment

**Figure 3:**
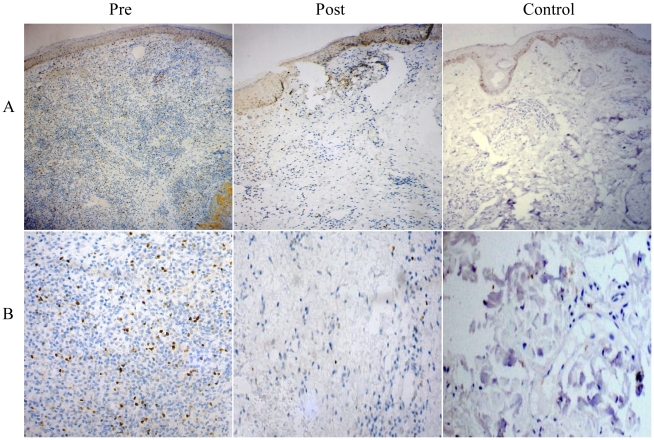
Immunohistochemical analysis of Foxp3 in tissue lesions of PKDL patients. Distribution of Foxp3 in dermal lesion tissue sections at pre treatment, post treatment stages and normal skin of healthy individuals. Panel A (10×), Panel B (40×) magnification.

To authenticate mRNA expression at protein level, IHC staining for Foxp3 expression was evaluated between groups. Three PKDL samples showed intense nuclear staining for Foxp3 in tissue infiltrate region. After treatment there was a substantial reduction in Foxp3^+^ cells and cell infiltrates. Representative examples are illustrated in Figure 3.

### Expression of Treg markers and IL-10 varied with clinical manifestation of disease

**Figure 4:**
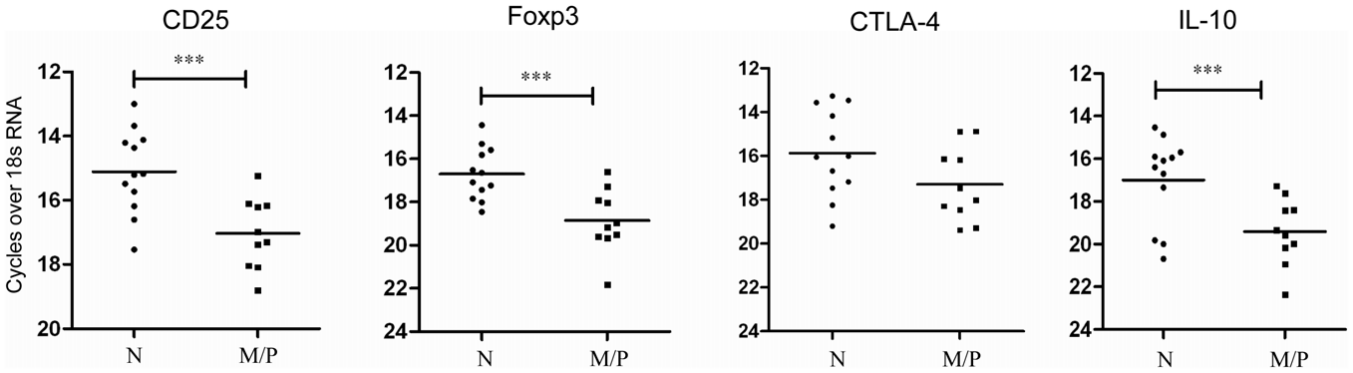
Ex vivo analysis of mRNA expression in different clinical manifestations of PKDL. *Ex vivo* analysis of relative Treg markers and IL-10 mRNA levels in Nodular (N) (n = 12) and Macular/Papular (M/P) (n = 10) lesions in distinct forms of PKDL. ***P<0.001.

We investigated preponderance of localized nTregs and IL-10 in nodular tissues compared to macular or papular clinical manifestations. mRNA analysis showed enhanced CD25, Foxp3 and IL-10 mRNA levels in nodular tissues compared to other forms (CD25 & Foxp3, *P*<0.001; IL-10, *P* = 0.006) (Figure 4).

## Discussion

During infection, a precise controlled immune response is desired to protect the host from invading parasites, preventing untoward immune responses and maintaining homeostasis. nTreg constitute one such arm of the regulatory network acting as a double edged sword, on one hand limiting inflammation mediated pathology, at the same time promoting parasite replication [8]. Recently we have reported a rich parasite burden in nodular tissue of PKDL patients compared to other forms. Further, we also showed positive correlation between circulating IL-10 and parasite burden in human VL [5]. Here we provide evidence for accumulation of nTreg in PKDL lesion tissues and demonstrate a direct correlation between CD25, Foxp3 and IL-10 mRNA levels with parasite load.

At the pre-treatment stage the expression of nTreg cells surface markers (CD25, CTLA-4 and Foxp3) was found elevated in lesion tissue compared to control, implicating a role of nTreg in PKDL. Reports on human CL have documented a possible role of intra-lesional Treg cells for local control of effector T cell functions and correlation with drug unresponsiveness [9], [10]. In contrast to PKDL, human VL was not linked with nTreg accumulation in blood or spleen, nor was antigen-specific IFN-γ response rescued following depletion of CD25^+^ cells [6]. Possible discrepancy between VL and PKDL could be context dependent due to (i) different niche and clinical manifestation; (ii) infection induced inflammation (iii) presence of Treg inducing and proliferating factors.

Of the human CD4^+^ T cells, approximately 30% cells express CD25. 1–3% of these express CD25 at high levels (CD25^++^) that possess suppressor activity [11]. The present study demonstrated elevated mRNA expression of CD25 in lesion tissue of patients and the level subsided after treatment, suggesting accumulation of Treg in lesion tissues supported by immunohistochemical identification of Foxp3+ cells. In Sudanese PKDL, scanty CD25+ cells in tissue biopsy are documented [12], contrary to this we noticed enhanced CD25 expression in all patients, which could be due to differences in the ethnic composition of the two populations or strains of the parasites. Thus, the finding suggests the variation in regulatory cells population according to the immune environment or the degree of inflammation and also suggests a distinct PKDL pathology in comparison with Indian VL or Sudanese PKDL.

The correlation between advent of host immune responses and parasite persistence has been demonstrated in various *Leishmania* infections [5], [13], [14]. Analysis of Foxp3 and CD25 mRNA levels and parasite load showed direct correlation in lesion tissues. Furthermore, nodular lesions have rich parasite burden compared to other forms, indicating that chronic infection in the skin might have generated a population of Treg cells that have influence on parasite propagation and the level of immunity. Numerous recent observations have shown influence of nTreg on functional immunity against several microbes including human malaria parasite [15].

Similar correlation with parasite load was lacking for CTLA-4, although mRNA was enhanced at pretreatment stage. CTLA-4 (CD152) is expressed on activated CD4+ and CD8+ T cells and binds to the costimulatory ligands, B7-1 (CD80) and B7-2 (CD86), with a 20-fold higher affinity than CD28 [16], [17]. CTLA-4 expression is not detected on naive T cells, but transcriptionally induced after T cell activation [18]. CTLA-4 can out-compete CD28 for binding with co-stimulatory ligands, especially when these molecules are limiting, and low level of CD28 expression on circulating T cells is reported in PKDL [19]. Thus, in the context of enhanced CTLA-4 at pretreatment stage, data suggests inhibitory local signals in lesion tissues. In murine VL, blockage of CTLA-4 results in beneficial effect, in the form of low parasite burden and increment in the frequency of IFN-γ and IL-4 producing cells in spleen and liver [20]. Further studies are needed to understand the extent to which CTLA-4 contributes its effect either on regulatory T cells or activated Th1 cells or on both and if blockage of CTLA-4 has any influence on functional immunity in human PKDL.

IL-10 is repeatedly implicated as an immunosuppressive factor in both human and experimental leishmaniasis. The role of IL-10 in KA and PKDL is well documented [4], [5] and a consistent correlation between IL-10 levels and the development of PKDL in Sudanese KA patients has been established [21]. Like nTreg markers, IL-10 mRNA level was enhanced in PKDL lesion tissues at pretreatment stage, the level subsided following treatment, similar to previous observations [4], [6]. Additionally, like Foxp3 and CD25, IL-10 mRNA level directly correlated with parasite burden, and was high in nodular lesions compared with other form, probably due to preponderance of nTreg and other cellular infiltrates in nodular lesions. Furthermore, in mice IL-10 is found to be important for the maintenance of Treg activity [22].

Immunopathology of different clinical manifestations of PKDL is not well understood. There is higher cellular infiltration including T cells and plasma cells in nodular PKDL compared to macular or papular forms [23], [24]. In addition, we have recently demonstrated [5], and also observed in the present study, a higher parasite burden in nodular lesions compared to macular or papular lesions. Abundance of nTreg with IL-10 in tissue infiltrates of nodular form, observed in the present study, may be the driving factors for high parasite burden resulting in disease aggravation.

Collectively the data suggests a possible role of Treg and IL-10 in parasite establishment in PKDL patients. Because nTreg have been shown to produce IL-10 and TGF-β associated with immune suppression in experimental systems, studies are warranted to explore antigen specific IL-10 source in PKDL lesion tissues. Furthermore functional studies are required to support the association of Treg and immunosuppression in PKDL. Such findings will lead to new targets for immunotherapeutic or vaccine strategies against PKDL, important from the perspective of parasite reservoir/transmission and a barrier towards VL eradication.
